# Metagenomic data-mining reveals contrasting microbial populations responsible for trimethylamine formation in human gut and marine ecosystems

**DOI:** 10.1099/mgen.0.000080

**Published:** 2016-09-20

**Authors:** Eleanor Jameson, Andrew C. Doxey, Ruth Airs, Kevin J. Purdy, J. Colin Murrell, Yin Chen

**Affiliations:** ^1^​ School of Life Sciences, Gibbet Hill Campus, The University of Warwick, Coventry, CV4 7AL, UK; ^2^​ Department of Biology, University of Waterloo, 200 University Ave. W, Waterloo, Ontario N2L 3G1, Canada; ^3^​ Plymouth Marine Laboratory, Prospect Pl, Plymouth PL1 3DH, UK; ^4^​ University of East Anglia, Norwich Research Park, Norwich NR4 7TJ, UK

**Keywords:** Trimethylamine, Marine, gut microbiome, metagenome

## Abstract

Existing metagenome datasets from many different environments contain untapped potential for understanding metabolic pathways and their biological impact. Our interest lies in the formation of trimethylamine (TMA), a key metabolite in both human health and climate change. Here, we focus on bacterial degradation pathways for choline, carnitine, glycine betaine and trimethylamine *N*-oxide (TMAO) to TMA in human gut and marine metagenomes. We found the TMAO reductase pathway was the most prevalent pathway in both environments. *Proteobacteria* were found to contribute the majority of the TMAO reductase pathway sequences, except in the stressed gut, where *Actinobacteria* dominated. Interestingly, in the human gut metagenomes, a high proportion of the *Proteobacteria* hits were accounted for by the genera *Klebsiella* and *Escherichia.* Furthermore* Klebsiella* and *Escherichia* harboured three of the four potential TMA-production pathways (choline, carnitine and TMAO), suggesting they have a key role in TMA cycling in the human gut. In addition to the intensive TMAO–TMA cycling in the marine environment, our data suggest that carnitine-to-TMA transformation plays an overlooked role in aerobic marine surface waters, whereas choline-to-TMA transformation is important in anaerobic marine sediments. Our study provides new insights into the potential key microbes and metabolic pathways for TMA formation in two contrasting environments.

## Data Summary

The metagenomes examined in this study were downloaded from CAMERA (now iMicrobe) and MG-RAST [Data citation 1–22] detailed in Table S1.

## Impact Statement

In this study we used the existing wealth of metagenome data to answer the question ‘which bacterial metabolic pathways are important in producing trimethylamine (TMA)?’ TMA has recently been demonstrated to play a vital role in both human health (linked to heart disease) and climate change (being a climate-active trace gas and precursor of the greenhouse gas methane). Previous studies have shown that both gut and marine sediment bacteria are capable of producing TMA, but no existing studies have looked at which bacterial genera or metabolic pathways contribute most. To this end we concentrated on two environments, where TMA-production has a critical impact, the human gut and marine environments. TMA is produced directly from choline, carnitine, glycine betaine (GBT) and trimethylamine-*N*-oxide (TMAO). Our analysis of both the human gut and marine environments revealed that the previously overlooked TMAO–TMA pathway was the most abundant, by utilizing a combination of blast and profile*-*HMM gene similarity searches of metagenome datasets. Our data indicate that the TMAO–TMA pathway has the greatest therapeutic potential as a target for improving human health and mitigating climate change.

## Introduction

In the last decade, meta-omics research has generated a wealth of data on the composition of microbial communities from diverse environments. We sought to use these data to analyze the distribution of microbial trimethylamine (TMA) formation pathways. In the human gut, TMA formation from choline and carnitine is linked to cardiovascular disease (CVD); through the hepatic formation of the proatherosclerosis compound, trimethylamine *N*-oxide [TMAO; (Wang *et al.*, 2011; Zhu *et al*., 2016; Koeth *et al.*, 2013)]. TMA also plays an essential role in marine ecosystems, being a major precursor (35–90 %) of the greenhouse gas methane in coastal sediments (King, 1984), and a major carbon and energy source in surface waters for the marine heterotroph clades of *Roseobacter* and SAR11 (Lidbury *et al.*, 2015; Sun *et al*., 2011). Although these disparate environments exhibit many fundamental differences, both the marine and human gut environments are subject to high osmotic stress. Furthermore marine sediments share low oxygen and high productivity with the gut. Whilst several marine sediment studies have evaluated which microbial species are involved in TMA formation (King, 1984,  1988), species information for gut TMA formation is lacking. Microorganisms in both marine and gut environments play essential roles in quaternary amine cycling and TMA-production, yet our understanding of the key microbes needs resolving. It leads us to ask which microorganisms and precursor molecules are key to TMA-production in these two contrasting ecosystems?

**Fig. 1.**
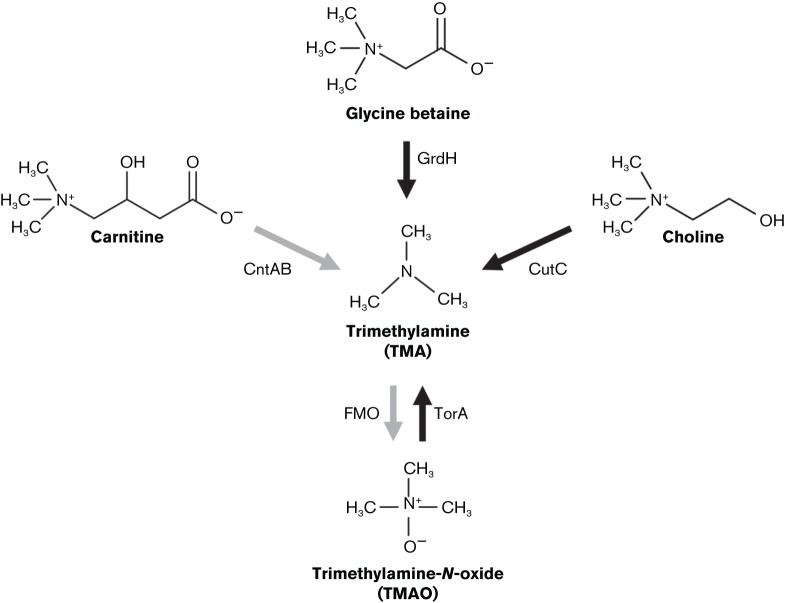
Direct formation pathways of trimethylamine (TMA). Genes encoding the key enzymes indicated were targeted for the data-mining. Key enzymes: CntAB, carnitine monooxygenase (Zhu *et al*., 2014); CutC, choline-TMA lyase (Craciun & Balskus, 2012); GrdH, glycine betaine reductase (Andreesen, 1994); TorA, trimethylamine *N*-oxide reductase (Méjean *et al*., 1994). Additionally the TMAO formation pathway FMO (flavin-containing monooxygenase) is indicated as it is critical to TMA cycling (Chen *et al.*, 2011). Black arrows denote anaerobic pathways and grey arrows denote aerobic pathways.

Several pathways for TMA formation are currently known (Fig. 1), involving choline–TMA lyase, CutC (Craciun & Balskus, 2012; Jameson *et al.*, 2015), carnitine monooxygenase, CntAB (Zhu *et al*., 2014), glycine betaine (GBT) reductase, GrdH (Andreesen, 1994), and additionally via the reduction of TMAO, TorA/TorZ/DorA (hereafter referred to as TorA; Méjean *et al.*, 1994; McCrindle *et al.*, 2005). Here we investigate the abundance of potential TMA-production pathways, through targeted datamining of human gut and marine metagenomes, offering new insights into the potential major precursors and key microbial players in TMA formation in these contrasting environments.

## Methods

We retrieved 9 human gut and 13 marine metagenome studies, comprising 221 datasets from public databases (Table S1, available in the online Supplementary Material), selected for their depth of coverage, in environments where TMA is important. The gut metagenomes were divided into “stressed” (ICU patient, pregnancy and extreme aging) and “healthy” (variety of diets, no reported illnesses). We combined blast and profile Hidden Markov Models (profile-HMM) methods to determine the abundance of potential TMA pathway genes. The *blastp* searches were conducted using blast+ (NCBI) with a single representative protein sequence query (Fig. S1, available in the online Supplementary Material), with an *E*-value cut off of 1×10^−5^. The representative protein sequence queries were selected because they had proven functions. The profile-HMMs used representative protein alignments of 10–30 reference sequences (Fig. S1) spanning each key TMA-pathway protein (Fig. 1). HMM-based metagenomic searches and taxonomic annotations were performed using the MetAnnotate pipeline (Petrenko *et al.*, 2015) with default parameters. Searches were performed using hmmsearch (HMMER 3.1b1) and USEARCH (Edgar, 2010) was used for best-hit classification, against the NCBI RefSeq database. Profile-HMMs attempted to negate the bias inherent in single sequence blast queries (Eddy, 2011). The resultant hits from both methods were used by MUSCLE 3.5 (Edgar, 2004) to build multiple protein alignments, then maximum likelihood phylogenetic trees were reconstructed using PhyML 3.0 (Guindon *et al.*, 2010). Phylogenetic mapping of these hit sequences against reference datasets (Fig. S1) allowed validation of the results with false-positive hits rejected. Positive hits were normalized to gene length.

## Results and Discussion

### Human gut

Results of previous studies have indicated that choline is the major precursor of TMA in the gut (Wang *et al.*, 2011; Craciun & Balskus, 2012; Tang *et al.*, 2013; Martínez-del Campo *et al.*, 2015; Ierardi *et al.*, 2015; Romano *et al.*, 2015). Our results support the idea of choline as the most important dietary contributor to TMA-production (e.g. higher than GrdH or CntA), however the TMAO (TorA-like) pathway had the highest detection rate. This high TorA-like abundance cannot be accounted for solely by dietary intake because TMAO is restricted primarily to marine fish (Mackay *et al.*, 2011; Svensson *et al.*, 1994). Alternatively we suggest an intensive cycling between TMA and TMAO within the gut environment, with TMAO being an important alternative electron receptor for anaerobic respiration by facultative gut microbiota (Winter *et al.*, 2013).

The glycine betaine (GrdH) pathway was detected at relatively low levels, which may be attributed to its requirement for the trace element, selenium, for enzyme activity (Freudenberg *et al.*, 1989). Clinical studies found a link between low plasma concentrations of selenium and CVD, while selenium supplementation trials did not improve outcomes (Flores-Mateo *et al*., 2006; Fairweather-Tait *et al.*, 2011); potentially indicating a selenium requirement by a CVD-inducing GrdH containing bacterial community (Freudenberg *et al.*, 1989). It is also likely that low abundance of GrdH in the gut metagenome was due to GBTs importance as a compatible solute. Accumulation of compatible solutes is necessary to combat stresses in the small intestine, such as volatile fatty acids, bile salts, high osmolarity and low oxygen (Sleator & Hill, 2002; Beumer *et al.*, 1994). *De novo* synthesis of compatible solutes, e.g. GBT and carnitine, are generally energy-expensive, therefore, their catabolism is likely to be rare in the gut, compounding the scarcity of the GrdH-like and CntA-like pathways. Additionally, the limited oxygen availability in the gut may contribute to the low abundance of the O_2_-dependent CntA pathway (Zhu *et al*., 2014).

**Fig. 2.**
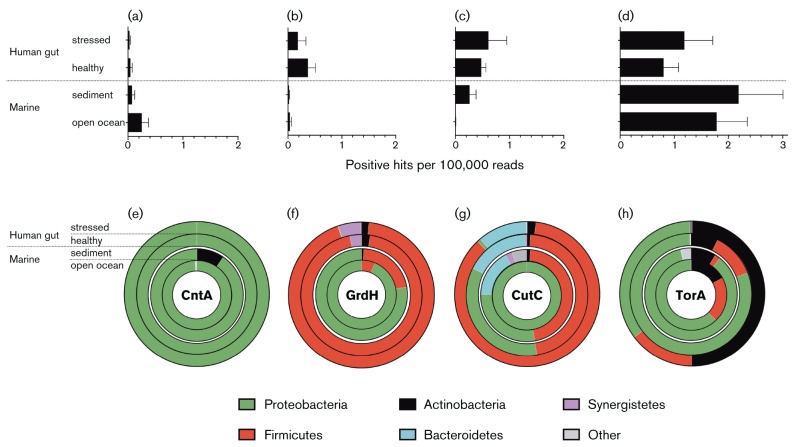
Data-mining of TMA pathway positive hits, combined* blastp* and profile-HMM searches of human gut and marine metagenomes. (a–d) represent positive, phylogenetically confirmed hits, normalized to gene length (CntA, 1116 bp; GrdH, 1314 bp; CutC, 3432 bp; TorA, 2529 bp). Error bars represent sem. Bar charts (a–d) represent relative abundance of hits: (a), CntA; (b), GrdH; (c), CutC; (d), TorA. Donut charts (e–h) shown the relative abundance (per 100 000 reads) of phylum-level classification of sequences obtained from blastp and profile-HMMs combined: (e), CntA; (f), GrdH; (g), CutC; (h), TorA. The outer rings represent stressed human gut (32 datasets); the second rings, represent healthy human gut (135 datasets); the third rings marine sediment (36 datasets) and the inner rings open ocean metagenomes (18 datasets; details in Table S1).

**Fig. 3.**
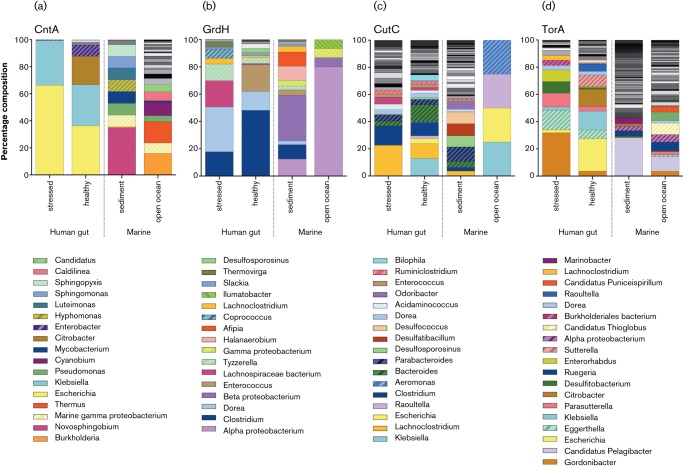
Bar charts depicting the genus-level assignments for confirmed hits. These illustrate relative percentage abundances of phylogenetically confirmed sequence hits at genus-level classification for sequences obtained from both *blastp* and profile-HMM combined.

The positive hits for the CntA and GrdH pathways were both dominated by single phyla,* Proteobacteria* and *Firmicutes*, respectively (Fig. 2e, f); however, key phylogenetic variations were observed between stressed and healthy gut datasets in CutC and TorA (Fig. 2g, h). For CutC 46 % hits were *Firmicutes* in the healthy gut, rising to 86 % in stressed datasets (Fig. 2g) whereas for TorA hits, *Actinobacteria* rose from 7 % in the healthy gut to 50 % in the stressed gut (Fig. 2h). Notably, at the genus level (Fig. 3), *Klebsiella* and *Escherichia* harboured three of the four potential TMA-production pathways, accounting for approximately 13 % and 4 % of CutC, approximately 30 % and 36 % of CntA and approximately 14 % and 24 % TorA in the healthy and stressed datasets, respectively.

### Marine

Paralleling the results from the gut, there was a high prevalence of TorA hits in the marine datasets indicating that TMAO also has a pivotal role in marine TMA cycling (Fig. 2a–d). This was somewhat surprising in the open-ocean since TMAO reduction is primarily considered an anaerobic pathway; however, the TorA enzyme has been shown to be active under aerobic conditions (Ansaldi *et al.*, 2007). TMAO formation from TMA can be attributed to widespread flavin-containing monooxygenase activity (Fig. 1) in a variety of marine biota (Chen *et al.*, 2011; Gibb & Hatton, 2004). As theorised for the gut, intensive TMA–TMAO cycling may also be important in marine systems.

The next most abundant TMA-production pathway varied between the marine sediment and open ocean datasets (Fig. 2). There appears to be a level of mutual exclusivity between the potential CntA- and CutC-pathways related to oxygen requirement, resulting in higher abundance of the CntA-like pathway in the aerobic open ocean and conversely the CutC-like pathway is more prevalent in anaerobic sediments (Fig. 2a–c).

The GrdH-like pathway was detected at the lowest abundances in the marine datasets, as described for the gut, and this could indicate the importance of GBT as a key compatible solute for marine microorganisms (Andreesen, 1994; Andreesen *et al.*, 1999).

Phylogenetically, all four genes showed dominance by *Proteobacteria* in the marine datasets, however *Firmicutes* made a significant contribution to GrdH- and CutC-hits in sediments and TorA-hits in open ocean datasets. At the genus level (Fig. 3), numerous diverse genera were detected, resulting in no notable overlaps of dominant genera between marine datasets, which is hardly surprisingly since sediments and the open oceans have distinct environmental characteristics.

## Conclusion

Our quantitative analyses of genes encoding TMA formation pathways in contrasting ecosystems imply that the TMAO reduction (TorA) pathway was the most prevalent TMA formation pathway in both the marine and gut environments, indicating intensive cycling between TMAO and TMA. These TorA-like enzymes have previously been detected in the human gut (Ravcheev & Thiele, 2014) and the marine environment (Dos Santos *et al*., 1998), but this cycle has been overlooked in TMA-related CVD studies (Tang & Hazen, 2014). With regard to diet-derived TMA, our results corroborate findings that choline is the most important dietary component (compared with GBT and carnitine), because CutC was the most abundant pathway for TMA formation in the gut. The anaerobic GrdH-like and aerobic CntA-like pathways were detected at the lowest levels across the environments, potentially due to their roles as compatible solutes (Andreesen *et al.*, 1999; Beumer *et al.*, 1994). The gut is largely anaerobic and this appeared to be reflected in the higher prevalence of the anaerobic pathways (TorA, CutC, GrdH). In the marine datasets, we split the metagenomes into low-oxygen sediments and high-oxygen open ocean, and these analyses suggest some mutual exclusion between oxygen-dependent carnitine and oxygen-free choline transformation to TMA.

## Supplementary Data

1643 Supplementary File 1
