# Supplementary material for: Metagenomic data-mining reveals contrasting microbial populations responsible for trimethylamine formation in human gut and marine ecosystems
Source: Microb Genom. 2016 Sep 20;2(9):e000080. doi: 10.1099/mgen.0.000080 (PMC5537630; doi:10.1099/mgen.0.000080)
Supplement: Supplementary File 1 [file mgen-02-80-s001.pdf]

A. Riske monoxygenase alpha subunit family

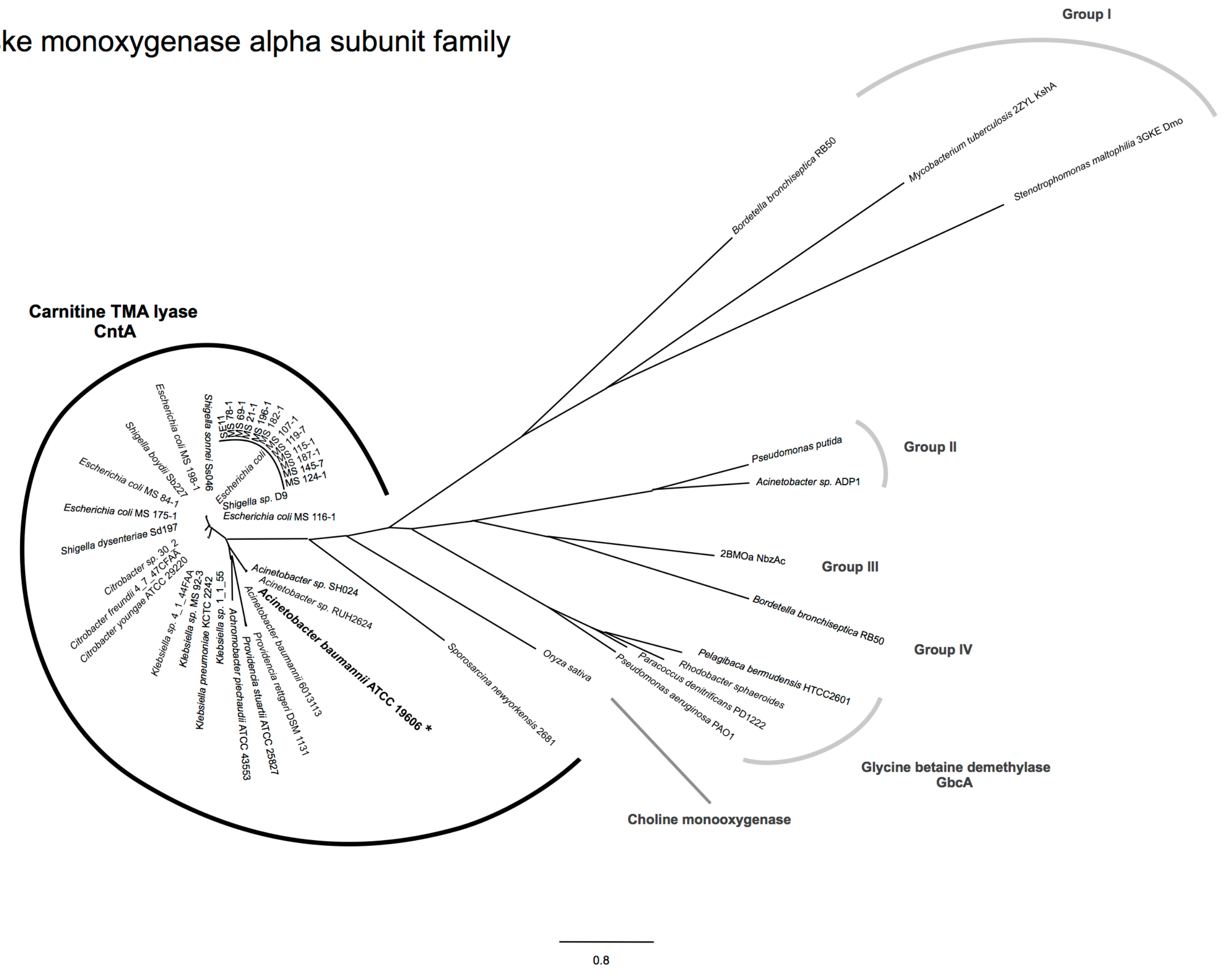

B. Glycine/betaine/sarcosine/D-proline reductase family

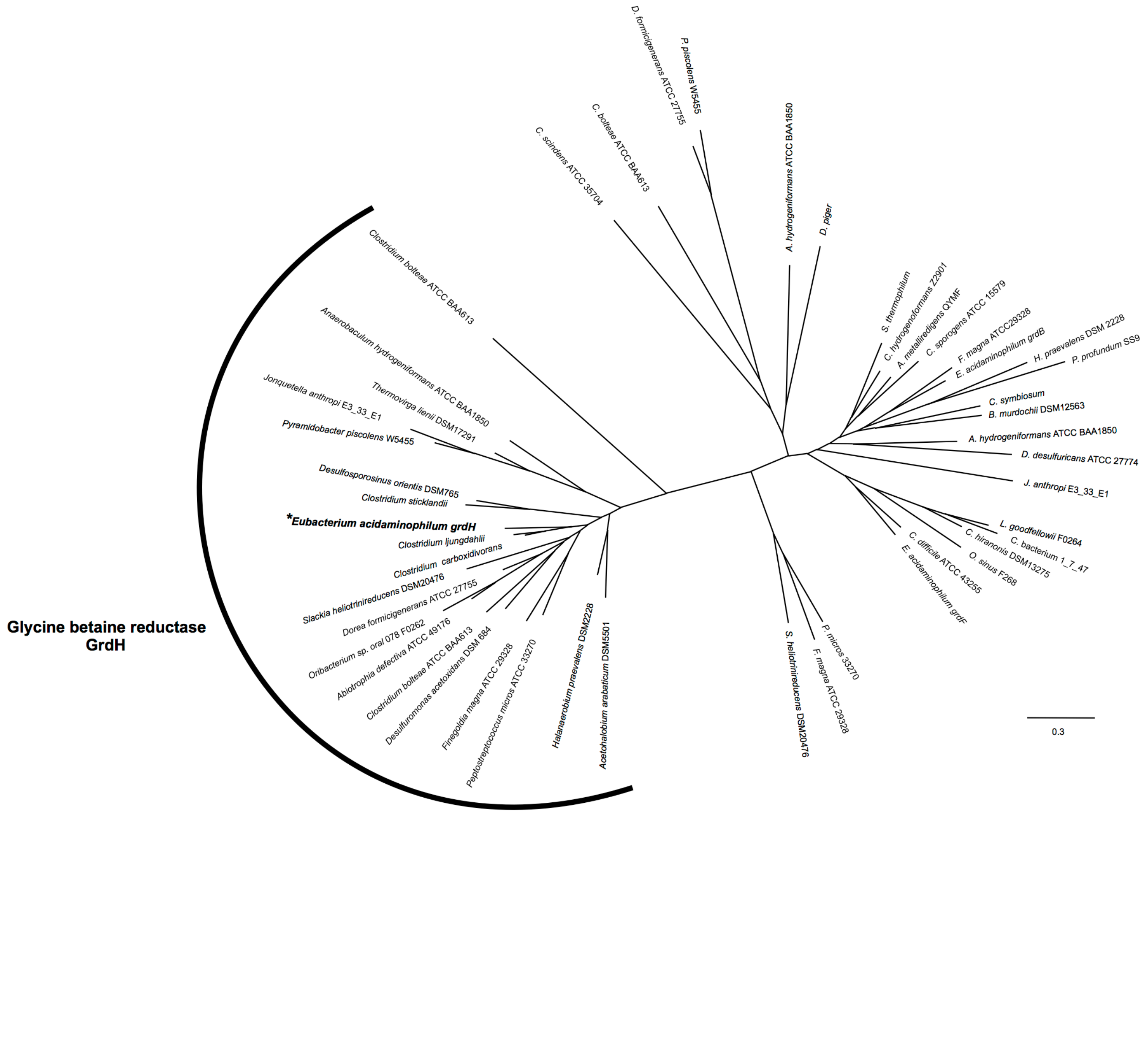

C. Pyruvate formate lyase family

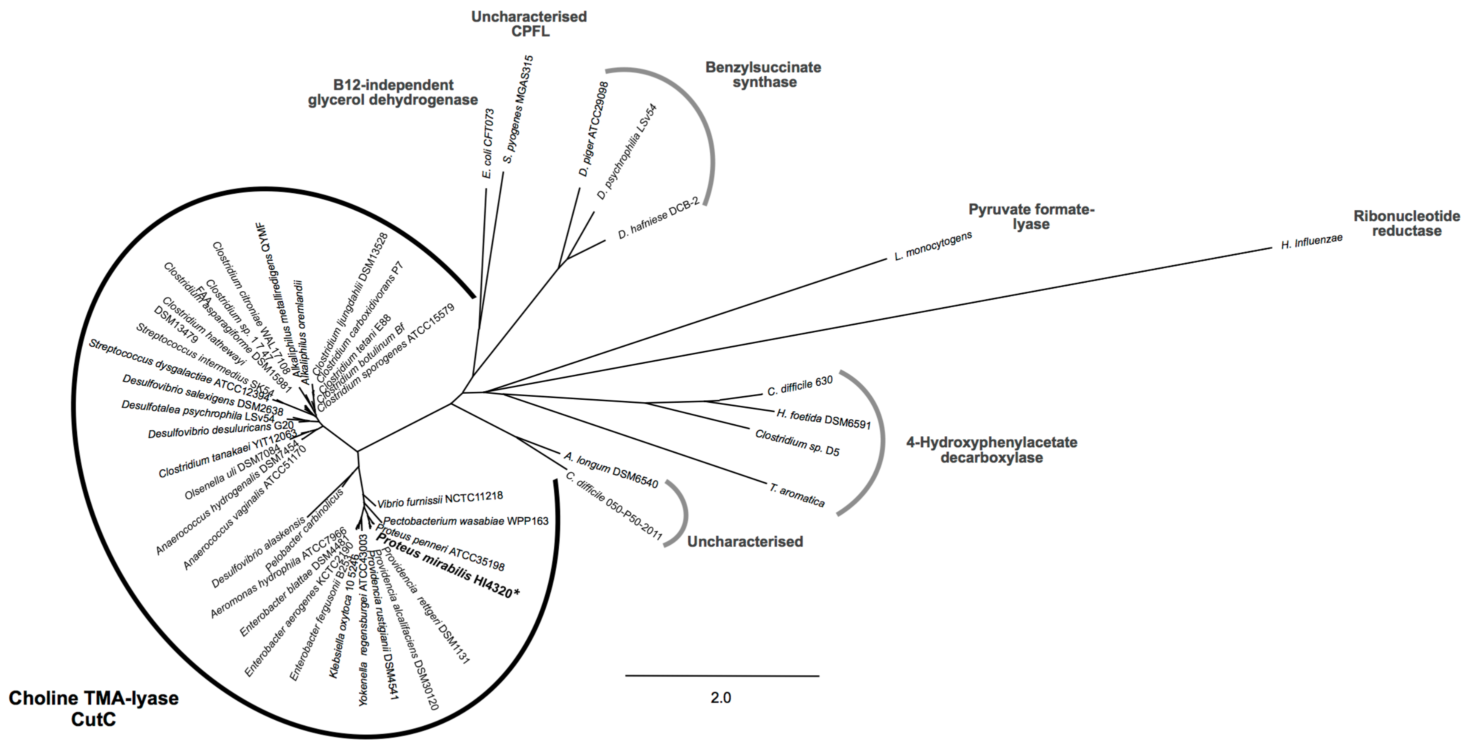

D. Molybdopterin oxidoreductase family

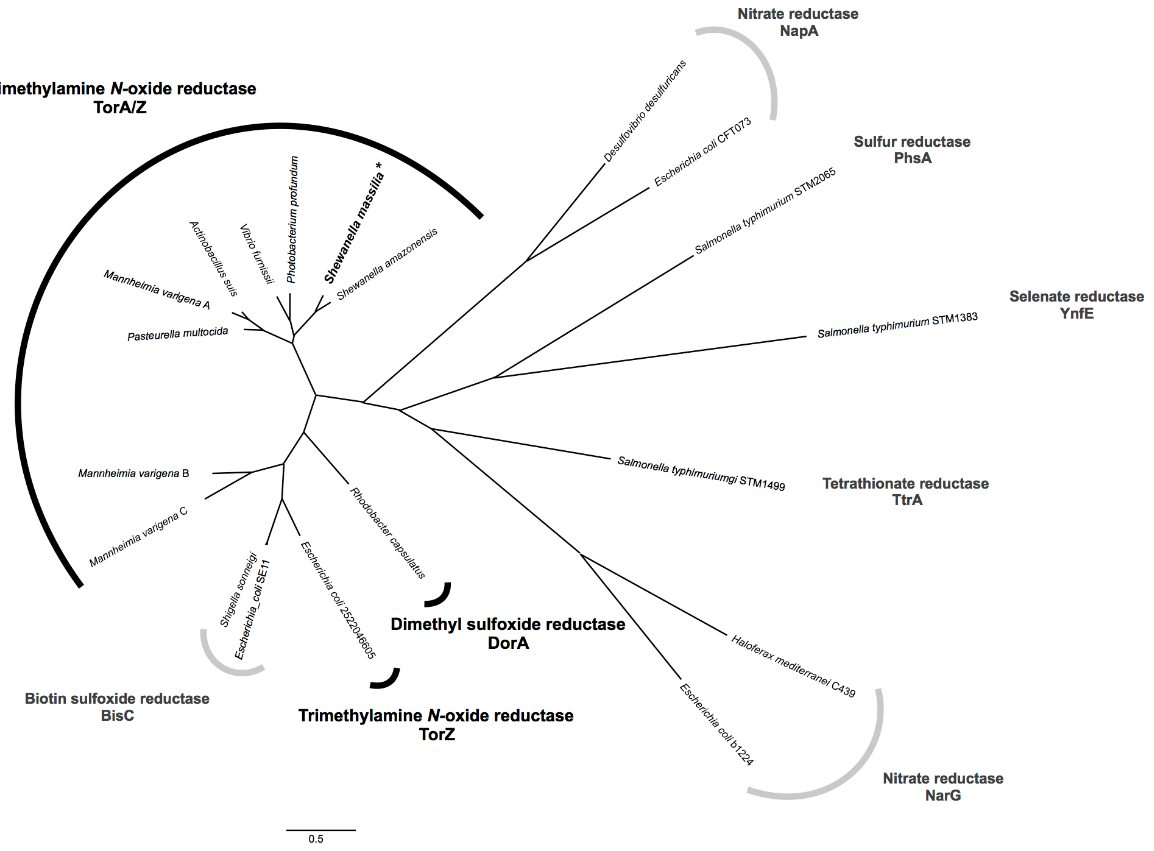

Environment	Metagenome study name	ID	Author	Project weblink	Date	Data weblink
Open ocean	North Pacific Metagenomes (CAMERA)	CAM_P_0001028_NorthPacificMetagenomes	Lin, Y.C., Campbell, T., Chung, C.C., Gong, G.C., Chiang, K.P. & Worden, A.Z.	North Pacific metagenomes from Monterey Bay to Open Ocean (CalCOFI Line 67) October 2007	2007	
Open ocean	Global Ocean Survey	GOS	Yutin, N., Suzuki, M.T., Teeling, H., Weber, M., Venter, J.C., Rusch, D.B. & Béjà, O	http://data.imicrobe.us/project/view/26	2008	http://data.imicrobe.us/project/view/26
Open ocean	Sapelo Island Bacterioplankton Vanillate/DSMP Study	4440358.3	Moran, M.A.	http://metagenomics.anl.gov/metagenomics.cgi?page=MetagenomeProject&project=19	2007	http://metagenomics.anl.gov/linkin.cgi?metagenome=4440358.3
Open ocean	Sapelo Island Bacterioplankton Vanillate/DSMP Study	4440360.3	Moran, M.A.	http://metagenomics.anl.gov/metagenomics.cgi?page=MetagenomeProject&project=19	2007	http://metagenomics.anl.gov/linkin.cgi?metagenome=4440360.3
Open ocean	Sapelo Island Bacterioplankton Vanillate/DSMP Study	4440361.3	Moran, M.A.	http://metagenomics.anl.gov/metagenomics.cgi?page=MetagenomeProject&project=19	2007	http://metagenomics.anl.gov/linkin.cgi?metagenome=4440361.3
Open ocean	Sapelo Island Bacterioplankton Vanillate/DSMP Study	4440362.3	Moran, M.A.	http://metagenomics.anl.gov/metagenomics.cgi?page=MetagenomeProject&project=19	2007	http://metagenomics.anl.gov/linkin.cgi?metagenome=4440362.3
Open ocean	Sapelo Island Bacterioplankton Vanillate/DSMP Study	4440363.3	Moran, M.A.	http://metagenomics.anl.gov/metagenomics.cgi?page=MetagenomeProject&project=19	2007	http://metagenomics.anl.gov/linkin.cgi?metagenome=4440363.3
Open ocean	Sapelo Island Bacterioplankton Metagenome	4443718.3	Moran, M.A.	http://metagenomics.anl.gov/metagenomics.cgi?page=MetagenomeProject&project=46	2007	http://metagenomics.anl.gov/linkin.cgi?metagenome=4443721.3
Open ocean	Sapelo Island Bacterioplankton Metagenome	4443719.3	Moran, M.A.	http://metagenomics.anl.gov/metagenomics.cgi?page=MetagenomeProject&project=46	2007	http://metagenomics.anl.gov/linkin.cgi?metagenome=4443719.3
Open ocean	Sapelo Island Bacterioplankton Metagenome	4443720.3	Moran, M.A.	http://metagenomics.anl.gov/metagenomics.cgi?page=MetagenomeProject&project=46	2007	http://metagenomics.anl.gov/linkin.cgi?metagenome=4443720.3
Open ocean	Sapelo Island Bacterioplankton Metagenome	4443722.3	Moran, M.A.	http://metagenomics.anl.gov/metagenomics.cgi?page=MetagenomeProject&project=46	2007	http://metagenomics.anl.gov/linkin.cgi?metagenome=4443722.3
Open ocean	Sapelo Island Bacterioplankton Metagenome	4443726.3	Moran, M.A.	http://metagenomics.anl.gov/metagenomics.cgi?page=MetagenomeProject&project=46	2007	http://metagenomics.anl.gov/linkin.cgi?metagenome=4443726.3
Open ocean	Monterey Bay Microbial Study	4443712.3	Delong, E.	http://metagenomics.anl.gov/metagenomics.cgi?page=MetagenomeProject&project=38	2010	http://metagenomics.anl.gov/linkin.cgi?metagenome=4443712.3
Open ocean	Monterey Bay Microbial Study	4443713.3	Delong, E.	http://metagenomics.anl.gov/metagenomics.cgi?page=MetagenomeProject&project=38	2010	http://metagenomics.anl.gov/linkin.cgi?metagenome=4443713.3
Open ocean	Monterey Bay Microbial Study	4443714.3	Delong, E.	http://metagenomics.anl.gov/metagenomics.cgi?page=MetagenomeProject&project=38	2010	http://metagenomics.anl.gov/linkin.cgi?metagenome=4443714.3
Open ocean	Monterey Bay Microbial Study	4443715.3	Delong, E.	http://metagenomics.anl.gov/metagenomics.cgi?page=MetagenomeProject&project=38	2010	http://metagenomics.anl.gov/linkin.cgi?metagenome=4443715.3
Open ocean	Monterey Bay Microbial Study	4443716.3	Delong, E.	http://metagenomics.anl.gov/metagenomics.cgi?page=MetagenomeProject&project=38	2010	http://metagenomics.anl.gov/linkin.cgi?metagenome=4443716.3
Open ocean	Monterey Bay Microbial Study	4443717.3	Delong, E.	http://metagenomics.anl.gov/metagenomics.cgi?page=MetagenomeProject&project=38	2010	http://metagenomics.anl.gov/linkin.cgi?metagenome=4443717.3
Marine sediment	Mangroves	4451033.3	Andreote, F.	http://metagenomics.anl.gov/metagenomics.cgi?page=MetagenomeProject&project=134	2011	http://metagenomics.anl.gov/linkin.cgi?metagenome=4451033.3
Marine sediment	Mangroves	4451034.3	Andreote, F.	http://metagenomics.anl.gov/metagenomics.cgi?page=MetagenomeProject&project=134	2011	http://metagenomics.anl.gov/linkin.cgi?metagenome=4451034.3
Marine sediment	Mangroves	4451035.3	Andreote, F.	http://metagenomics.anl.gov/metagenomics.cgi?page=MetagenomeProject&project=134	2011	http://metagenomics.anl.gov/linkin.cgi?metagenome=4451035.3
Marine sediment	Mangroves	4451036.3	Andreote, F.	http://metagenomics.anl.gov/metagenomics.cgi?page=MetagenomeProject&project=134	2011	http://metagenomics.anl.gov/linkin.cgi?metagenome=4451036.3
Marine sediment	Mangrove RJ BA	4485218.3	Vasconcelos, A.T.	http://metagenomics.anl.gov/metagenomics.cgi?page=MetagenomeProject&project=6544	2013	http://metagenomics.anl.gov/linkin.cgi?metagenome=4485218.3
Marine sediment	Mangrove RJ BA	4485219.3	Vasconcelos, A.T.	http://metagenomics.anl.gov/metagenomics.cgi?page=MetagenomeProject&project=6544	2013	http://metagenomics.anl.gov/linkin.cgi?metagenome=4485219.3
Marine sediment	Mangrove RJ BA	4485981.3	Vasconcelos, A.T.	http://metagenomics.anl.gov/metagenomics.cgi?page=MetagenomeProject&project=6544	2013	http://metagenomics.anl.gov/linkin.cgi?metagenome=4485981.3
Marine sediment	Mangrove RJ BA	4485982.3	Vasconcelos, A.T.	http://metagenomics.anl.gov/metagenomics.cgi?page=MetagenomeProject&project=6544	2013	http://metagenomics.anl.gov/linkin.cgi?metagenome=4485982.3
Marine sediment	Peru OMZ - Meteor Cruise M77/3	4450891.3	LaRoche, J.	http://metagenomics.anl.gov/metagenomics.cgi?page=MetagenomeProject&project=6556	2013	http://metagenomics.anl.gov/linkin.cgi?metagenome=4450891.3
Marine sediment	Peru OMZ - Meteor Cruise M77/3	4450892.3	LaRoche, J.	http://metagenomics.anl.gov/metagenomics.cgi?page=MetagenomeProject&project=6556	2013	http://metagenomics.anl.gov/linkin.cgi?metagenome=4450892.3
Marine sediment	Peru OMZ - Meteor Cruise M77/3	4452042.3	LaRoche, J.	http://metagenomics.anl.gov/metagenomics.cgi?page=MetagenomeProject&project=6556	2013	http://metagenomics.anl.gov/linkin.cgi?metagenome=4452042.3
Marine sediment	Peru OMZ - Meteor Cruise M77/3	4452043.3	LaRoche, J.	http://metagenomics.anl.gov/metagenomics.cgi?page=MetagenomeProject&project=6556	2013	http://metagenomics.anl.gov/linkin.cgi?metagenome=4452043.3
Marine sediment	Peru OMZ - Meteor Cruise M77/3	4460676.3	LaRoche, J.	http://metagenomics.anl.gov/metagenomics.cgi?page=MetagenomeProject&project=6556	2013	http://metagenomics.anl.gov/linkin.cgi?metagenome=4460676.3
Marine sediment	Peru OMZ - Meteor Cruise M77/3	4460677.3	LaRoche, J.	http://metagenomics.anl.gov/metagenomics.cgi?page=MetagenomeProject&project=6556	2013	http://metagenomics.anl.gov/linkin.cgi?metagenome=4460677.3
Marine sediment	Peru OMZ - Meteor Cruise M77/3	4460735.3	LaRoche, J.	http://metagenomics.anl.gov/metagenomics.cgi?page=MetagenomeProject&project=6556	2013	http://metagenomics.anl.gov/linkin.cgi?metagenome=4460735.3
Marine sediment	Peru OMZ - Meteor Cruise M77/3	4460736.3	LaRoche, J.	http://metagenomics.anl.gov/metagenomics.cgi?page=MetagenomeProject&project=6556	2013	http://metagenomics.anl.gov/linkin.cgi?metagenome=4460736.3
Marine sediment	Peru OMZ - Meteor Cruise M77/3	4461588.3	LaRoche, J.	http://metagenomics.anl.gov/metagenomics.cgi?page=MetagenomeProject&project=6556	2013	http://metagenomics.anl.gov/linkin.cgi?metagenome=4461588.3
Marine sediment	Peru OMZ - Meteor Cruise M77/3	4461589.3	LaRoche, J.	http://metagenomics.anl.gov/metagenomics.cgi?page=MetagenomeProject&project=6556	2013	http://metagenomics.anl.gov/linkin.cgi?metagenome=4461589.3
Marine sediment	Peru OMZ - Meteor Cruise M77/3	4462177.3	LaRoche, J.	http://metagenomics.anl.gov/metagenomics.cgi?page=MetagenomeProject&project=6556	2013	http://metagenomics.anl.gov/linkin.cgi?metagenome=4462177.3
Marine sediment	Peru Margin Subseafloor Biosphere	4459940.3	Biddle, J.F.	http://metagenomics.anl.gov/metagenomics.cgi?page=MetagenomeProject&project=105	2013	http://metagenomics.anl.gov/linkin.cgi?metagenome=4459940.3
Marine sediment	Peru Margin Subseafloor Biosphere	4459941.3	Biddle, J.F.	http://metagenomics.anl.gov/metagenomics.cgi?page=MetagenomeProject&project=105	2013	http://metagenomics.anl.gov/linkin.cgi?metagenome=4459941.3
Marine sediment	Peru Margin Subseafloor Biosphere	4440960.3	Biddle, J.F.	http://metagenomics.anl.gov/metagenomics.cgi?page=MetagenomeProject&project=105	2013	http://metagenomics.anl.gov/linkin.cgi?metagenome=4440960.3
Marine sediment	Peru Margin Subseafloor Biosphere	4440961.3	Biddle, J.F.	http://metagenomics.anl.gov/metagenomics.cgi?page=MetagenomeProject&project=105	2013	http://metagenomics.anl.gov/linkin.cgi?metagenome=4440961.3
Marine sediment	Peru Margin Subseafloor Biosphere	4440973.3	Biddle, J.F.	http://metagenomics.anl.gov/metagenomics.cgi?page=MetagenomeProject&project=105	2013	http://metagenomics.anl.gov/linkin.cgi?metagenome=4440973.3
Marine sediment	Subsurface Rifle	4441834.3	Meyer, F.	http://metagenomics.anl.gov/metagenomics.cgi?page=MetagenomeProject&project=119	2007	http://metagenomics.anl.gov/linkin.cgi?metagenome=4441834.3
Marine sediment	Subsurface Rifle	4441835.3	Meyer, F.	http://metagenomics.anl.gov/metagenomics.cgi?page=MetagenomeProject&project=119	2007	http://metagenomics.anl.gov/linkin.cgi?metagenome=4441835.3
Marine sediment	Subsurface Rifle	4442086.3	Meyer, F.	http://metagenomics.anl.gov/metagenomics.cgi?page=MetagenomeProject&project=119	2007	http://metagenomics.anl.gov/linkin.cgi?metagenome=4442086.3
Marine sediment	Subsurface Rifle	4450653.3	Meyer, F.	http://metagenomics.anl.gov/metagenomics.cgi?page=MetagenomeProject&project=119	2007	http://metagenomics.anl.gov/linkin.cgi?metagenome=4450653.3
Marine sediment	Subsurface Rifle	4465820.3	Meyer, F.	http://metagenomics.anl.gov/metagenomics.cgi?page=MetagenomeProject&project=119	2007	http://metagenomics.anl.gov/linkin.cgi?metagenome=4465820.3
Marine sediment	Subsurface Rifle	4465822.3	Meyer, F.	http://metagenomics.anl.gov/metagenomics.cgi?page=MetagenomeProject&project=119	2007	http://metagenomics.anl.gov/linkin.cgi?metagenome=4465822.3
Marine sediment	Subsurface Rifle	4465823.3	Meyer, F.	http://metagenomics.anl.gov/metagenomics.cgi?page=MetagenomeProject&project=119	2013	http://metagenomics.anl.gov/linkin.cgi?metagenome=4465823.3
Marine sediment	pink berry consortia - Illumina MiSeq	4516362.3	Wilbanks, E.	http://metagenomics.anl.gov/metagenomics.cgi?page=MetagenomeProject&project=3755	2014	http://metagenomics.anl.gov/linkin.cgi?metagenome=4516362.3
Marine sediment	pink berry consortia - Illumina HiSeq - ncbi SRA SRX332174	4517592.3	Wilbanks, E.	http://metagenomics.anl.gov/metagenomics.cgi?page=MetagenomeProject&project=3755	2014	http://metagenomics.anl.gov/linkin.cgi?metagenome=4517592.3
Marine sediment	Sweetwater wetlands	4517766.3	Parker, R.	http://metagenomics.anl.gov/metagenomics.cgi?page=MetagenomeProject&project=3910	2013	http://metagenomics.anl.gov/linkin.cgi?metagenome=4517766.3
Marine sediment	MaCuMBA tidal pool	4548349.3	Stal, L.	http://metagenomics.anl.gov/metagenomics.cgi?page=MetagenomeProject&project=7332	2013	http://metagenomics.anl.gov/linkin.cgi?metagenome=4548349.3
Marine sediment	MaCuMBA tidal pool	4548350.3	Stal, L.	http://metagenomics.anl.gov/metagenomics.cgi?page=MetagenomeProject&project=7332	2013	http://metagenomics.anl.gov/linkin.cgi?metagenome=4548350.3
Human gut - healthy	Human gut metagenome	13 Healthy Human Gut Metagenomes (JPN)	Hayashi, T. & Hattori, M.	http://data.imicrobe.us/project/view/33	2007	
Human gut - healthy	Twin Gut Microflora Study	4440452.7	Gordon, J.	http://metagenomics.anl.gov/metagenomics.cgi?page=MetagenomeProject&project=10	2008	http://metagenomics.anl.gov/linkin.cgi?metagenome=4440452.7
Human gut - healthy	Twin Gut Microflora Study	4440453.6	Gordon, J.	http://metagenomics.anl.gov/metagenomics.cgi?page=MetagenomeProject&project=10	2008	http://metagenomics.anl.gov/linkin.cgi?metagenome=4440453.6
Human gut - healthy	Twin Gut Microflora Study	4440460.5	Gordon, J.	http://metagenomics.anl.gov/metagenomics.cgi?page=MetagenomeProject&project=10	2008	http://metagenomics.anl.gov/linkin.cgi?metagenome=4440460.5
Human gut - healthy	Twin Gut Microflora Study	4440461.5	Gordon, J.	http://metagenomics.anl.gov/metagenomics.cgi?page=MetagenomeProject&project=10	2008	http://metagenomics.anl.gov/linkin.cgi?metagenome=4440461.5
Human gut - healthy	Twin Gut Microflora Study	4440462.5	Gordon, J.	http://metagenomics.anl.gov/metagenomics.cgi?page=MetagenomeProject&project=10	2008	http://metagenomics.anl.gov/linkin.cgi?metagenome=4440462.5
Human gut - healthy	Twin Gut Microflora Study	4440595.4	Gordon, J.	http://metagenomics.anl.gov/metagenomics.cgi?page=MetagenomeProject&project=10	2008	http://metagenomics.anl.gov/linkin.cgi?metagenome=4440595.4
Human gut - healthy	Twin Gut Microflora Study	4440610.3	Gordon, J.	http://metagenomics.anl.gov/metagenomics.cgi?page=MetagenomeProject&project=10	2008	http://metagenomics.anl.gov/linkin.cgi?metagenome=4440610.3
Human gut - healthy	Twin Gut Microflora Study	4440611.3	Gordon, J.	http://metagenomics.anl.gov/metagenomics.cgi?page=MetagenomeProject&project=10	2008	http://metagenomics.anl.gov/linkin.cgi?metagenome=4440611.3
Human gut - healthy	Twin Gut Microflora Study	4440613.3	Gordon, J.	http://metagenomics.anl.gov/metagenomics.cgi?page=MetagenomeProject&project=10	2008	http://metagenomics.anl.gov/linkin.cgi?metagenome=4440613.3
Human gut - healthy	Twin Gut Microflora Study	4440614.3	Gordon, J.	http://metagenomics.anl.gov/metagenomics.cgi?page=MetagenomeProject&project=10	2008	http://metagenomics.anl.gov/linkin.cgi?metagenome=4440614.3
Human gut - healthy	Twin Gut Microflora Study	4440615.3	Gordon, J.	http://metagenomics.anl.gov/metagenomics.cgi?page=MetagenomeProject&project=10	2008	http://metagenomics.anl.gov/linkin.cgi?metagenome=4440615.3
Human gut - healthy	Twin Gut Microflora Study	4440616.3	Gordon, J.	http://metagenomics.anl.gov/metagenomics.cgi?page=MetagenomeProject&project=10	2008	http://metagenomics.anl.gov/linkin.cgi?metagenome=4440616.3
Human gut - healthy	Twin Gut Microflora Study	4440639.3	Gordon, J.	http://metagenomics.anl.gov/metagenomics.cgi?page=MetagenomeProject&project=10	2008	http://metagenomics.anl.gov/linkin.cgi?metagenome=4440639.3
Human gut - healthy	Twin Gut Microflora Study	4440640.3	Gordon, J.	http://metagenomics.anl.gov/metagenomics.cgi?page=MetagenomeProject&project=10	2008	http://metagenomics.anl.gov/linkin.cgi?metagenome=4440640.3
Human gut - healthy	Twin Gut Microflora Study	4440823.3	Gordon, J.	http://metagenomics.anl.gov/metagenomics.cgi?page=MetagenomeProject&project=10	2008	http://metagenomics.anl.gov/linkin.cgi?metagenome=4440823.3
Human gut - healthy	Twin Gut Microflora Study	4440824.3	Gordon, J.	http://metagenomics.anl.gov/metagenomics.cgi?page=MetagenomeProject&project=10	2008	http://metagenomics.anl.gov/linkin.cgi?metagenome=4440824.3
Human gut - healthy	Twin Gut Microflora Study	4440825.3	Gordon, J.	http://metagenomics.anl.gov/metagenomics.cgi?page=MetagenomeProject&project=10	2008	http://metagenomics.anl.gov/linkin.cgi?metagenome=4440825.3
Human gut - healthy	Twin Gut Microflora Study	4440826.3	Gordon, J.	http://metagenomics.anl.gov/metagenomics.cgi?page=MetagenomeProject&project=10	2008	http://metagenomics.anl.gov/linkin.cgi?metagenome=4440826.3
Human gut - healthy	Human Faeces – Kurokawa	4440940.3	Hattori, M.	http://metagenomics.anl.gov/metagenomics.cgi?page=MetagenomeProject&project=29	2007	http://metagenomics.anl.gov/linkin.cgi?metagenome=4440940.3
Human gut - healthy	Human Faeces – Kurokawa	4440941.3	Hattori, M.	http://metagenomics.anl.gov/metagenomics.cgi?page=MetagenomeProject&project=29	2007	http://metagenomics.anl.gov/linkin.cgi?metagenome=4440941.3
Human gut - healthy	Human Faeces – Kurokawa	4440948.3	Hattori, M.	http://metagenomics.anl.gov/metagenomics.cgi?page=MetagenomeProject&project=29	2007	http://metagenomics.anl.gov/linkin.cgi?metagenome=4440948.3
Human gut - healthy	Human gut microbiome viewed across age and geography (WGS)	4461119.3	Gordon, J.	http://metagenomics.anl.gov/metagenomics.cgi?page=MetagenomeProject&project=98	2012	http://metagenomics.anl.gov/linkin.cgi?metagenome=4461119.3
Human gut - healthy	Human gut microbiome viewed across age and geography (WGS)	4461120.3	Gordon, J.	http://metagenomics.anl.gov/metagenomics.cgi?page=MetagenomeProject&project=98	2012	http://metagenomics.anl.gov/linkin.cgi?metagenome=4461120.3
Human gut - healthy	Human gut microbiome viewed across age and geography (WGS)	4461122.3	Gordon, J.	http://metagenomics.anl.gov/metagenomics.cgi?page=MetagenomeProject&project=98	2012	http://metagenomics.anl.gov/linkin.cgi?metagenome=4461122.3
Human gut - healthy	Human gut microbiome viewed across age and geography (WGS)	4461123.3	Gordon, J.	http://metagenomics.anl.gov/metagenomics.cgi?page=MetagenomeProject&project=98	2012	http://metagenomics.anl.gov/linkin.cgi?metagenome=4461123.3
Human gut - healthy	Human gut microbiome viewed across age and geography (WGS)	4461124.3	Gordon, J.	http://metagenomics.anl.gov/metagenomics.cgi?page=MetagenomeProject&project=98	2012	http://metagenomics.anl.gov/linkin.cgi?metagenome=4461124.3
Human gut - healthy	Human gut microbiome viewed across age and geography (WGS)	4461125.3	Gordon, J.	http://metagenomics.anl.gov/metagenomics.cgi?page=MetagenomeProject&project=98	2012	http://metagenomics.anl.gov/linkin.cgi?metagenome=4461125.3
Human gut - healthy	Human gut microbiome viewed across age and geography (WGS)	4461126.3	Gordon, J.	http://metagenomics.anl.gov/metagenomics.cgi?page=MetagenomeProject&project=98	2012	http://metagenomics.anl.gov/linkin.cgi?metagenome=4461126.3
Human gut - healthy	Human gut microbiome viewed across age and geography (WGS)	4461127.3	Gordon, J.	http://metagenomics.anl.gov/metagenomics.cgi?page=MetagenomeProject&project=98	2012	http://metagenomics.anl.gov/linkin.cgi?metagenome=4461127.3
Human gut - healthy	Human gut microbiome viewed across age and geography (WGS)	4461129.3	Gordon, J.	http://metagenomics.anl.gov/metagenomics.cgi?page=MetagenomeProject&project=98	2012	http://metagenomics.anl.gov/linkin.cgi?metagenome=4461129.3
Human gut - healthy	Human gut microbiome viewed across age and geography (WGS)	4461130.3	Gordon, J.	http://metagenomics.anl.gov/metagenomics.cgi?page=MetagenomeProject&project=98	2012	http://metagenomics.anl.gov/linkin.cgi?metagenome=4461130.3
Human gut - healthy	Human gut microbiome viewed across age and geography (WGS)	4461131.3	Gordon, J.	http://metagenomics.anl.gov/metagenomics.cgi?page=MetagenomeProject&project=98	2012	http://metagenomics.anl.gov/linkin.cgi?metagenome=4461131.3
Human gut - healthy	Human gut microbiome viewed across age and geography (WGS)	4461133.3	Gordon, J.	http://metagenomics.anl.gov/metagenomics.cgi?page=MetagenomeProject&project=98	2012	http://metagenomics.anl.gov/linkin.cgi?metagenome=4461133.3
Human gut - healthy	Human gut microbiome viewed across age and geography (WGS)	4461134.3	Gordon, J.	http://metagenomics.anl.gov/metagenomics.cgi?page=MetagenomeProject&project=98	2012	http://metagenomics.anl.gov/linkin.cgi?metagenome=4461134.3
Human gut - healthy	Human gut microbiome viewed across age and geography (WGS)	4461135.3	Gordon, J.	http://metagenomics.anl.gov/metagenomics.cgi?page=MetagenomeProject&project=98	2012	http://metagenomics.anl.gov/linkin.cgi?metagenome=4461135.3
Human gut - healthy	Human gut microbiome viewed across age and geography (WGS)	4461136.3	Gordon, J.	http://metagenomics.anl.gov/metagenomics.cgi?page=MetagenomeProject&project=98	2012	http://metagenomics.anl.gov/linkin.cgi?metagenome=4461136.3
Human gut - healthy	Human gut microbiome viewed across age and geography (WGS)	4461137.3	Gordon, J.	http://metagenomics.anl.gov/metagenomics.cgi?page=MetagenomeProject&project=98	2012	http://metagenomics.anl.gov/linkin.cgi?metagenome=4461137.3
Human gut - healthy	Human gut microbiome viewed across age and geography (WGS)	4461138.3	Gordon, J.	http://metagenomics.anl.gov/metagenomics.cgi?page=MetagenomeProject&project=98	2012	http://metagenomics.anl.gov/linkin.cgi?metagenome=4461138.3
Human gut - healthy	Human gut microbiome viewed across age and geography (WGS)	4461139.3	Gordon, J.	http://metagenomics.anl.gov/metagenomics.cgi?page=MetagenomeProject&project=98	2012	http://metagenomics.anl.gov/linkin.cgi?metagenome=4461139.3
Human gut - healthy	Human gut microbiome viewed across age and geography (WGS)	4461140.3	Gordon, J.	http://metagenomics.anl.gov/metagenomics.cgi?page=MetagenomeProject&project=98	2012	http://metagenomics.anl.gov/linkin.cgi?metagenome=4461140.3
Human gut - healthy	Human gut microbiome viewed across age and geography (WGS)	4461141.3	Gordon, J.	http://metagenomics.anl.gov/metagenomics.cgi?page=MetagenomeProject&project=98	2012	http://metagenomics.anl.gov/linkin.cgi?metagenome=4461141.3
Human gut - healthy	Human gut microbiome viewed across age and geography (WGS)	4461142.3	Gordon, J.	http://metagenomics.anl.gov/metagenomics.cgi?page=MetagenomeProject&project=98	2012	http://metagenomics.anl.gov/linkin.cgi?metagenome=4461142.3
Human gut - healthy	Human gut microbiome viewed across age and geography (WGS)	4461143.3	Gordon, J.	http://metagenomics.anl.gov/metagenomics.cgi?page=MetagenomeProject&project=98	2012	http://metagenomics.anl.gov/linkin.cgi?metagenome=4461143.3
Human gut - healthy	Human gut microbiome viewed across age and geography (WGS)	4461144.3	Gordon, J.	http://metagenomics.anl.gov/metagenomics.cgi?page=MetagenomeProject&project=98	2012	http://metagenomics.anl.gov/linkin.cgi?metagenome=4461144.3
Human gut - healthy	Human gut microbiome viewed across age and geography (WGS)	4461145.3	Gordon, J.	http://metagenomics.anl.gov/metagenomics.cgi?page=MetagenomeProject&project=98	2012	http://metagenomics.anl.gov/linkin.cgi?metagenome=4461145.3
Human gut - healthy	Human gut microbiome viewed across age and geography (WGS)	4461146.3	Gordon, J.	http://metagenomics.anl.gov/metagenomics.cgi?page=MetagenomeProject&project=98	2012	http://metagenomics.anl.gov/linkin.cgi?metagenome=4461146.3
Human gut - healthy	Human gut microbiome viewed across age and geography (WGS)	4461147.3	Gordon, J.	http://metagenomics.anl.gov/metagenomics.cgi?page=MetagenomeProject&project=98	2012	http://metagenomics.anl.gov/linkin.cgi?metagenome=4461147.3
Human gut - healthy	Human gut microbiome viewed across age and geography (WGS)	4461148.3	Gordon, J.	http://metagenomics.anl.gov/metagenomics.cgi?page=MetagenomeProject&project=98	2012	http://metagenomics.anl.gov/linkin.cgi?metagenome=4461148.3
Human gut - healthy	Human gut microbiome viewed across age and geography (WGS)	4461149.3	Gordon, J.	http://metagenomics.anl.gov/metagenomics.cgi?page=MetagenomeProject&project=98	2012	http://metagenomics.anl.gov/linkin.cgi?metagenome=4461149.3
Human gut - healthy	Human gut microbiome viewed across age and geography (WGS)	4461150.3	Gordon, J.	http://metagenomics.anl.gov/metagenomics.cgi?page=MetagenomeProject&project=98	2012	http://metagenomics.anl.gov/linkin.cgi?metagenome=4461150.3
Human gut - healthy	Human gut microbiome viewed across age and geography (WGS)	4461151.3	Gordon, J.	http://metagenomics.anl.gov/metagenomics.cgi?page=MetagenomeProject&project=98	2012	http://metagenomics.anl.gov/linkin.cgi?metagenome=4461151.3
Human gut - healthy	Human gut microbiome viewed across age and geography (WGS)	4461153.3	Gordon, J.	http://metagenomics.anl.gov/metagenomics.cgi?page=MetagenomeProject&project=98	2012	http://metagenomics.anl.gov/linkin.cgi?metagenome=4461153.3
Human gut - healthy	Human gut microbiome viewed across age and geography (WGS)	4461154.3	Gordon, J.	http://metagenomics.anl.gov/metagenomics.cgi?page=MetagenomeProject&project=98	2012	http://metagenomics.anl.gov/linkin.cgi?metagenome=4461154.3
Human gut - healthy	Human gut microbiome viewed across age and geography (WGS)	4461155.3	Gordon, J.	http://metagenomics.anl.gov/metagenomics.cgi?page=MetagenomeProject&project=98	2012	http://metagenomics.anl.gov/linkin.cgi?metagenome=4461155.3
Human gut - healthy	Human gut microbiome viewed across age and geography (WGS)	4461159.3	Gordon, J.	http://metagenomics.anl.gov/metagenomics.cgi?page=MetagenomeProject&project=98	2012	http://metagenomics.anl.gov/linkin.cgi?metagenome=4461159.3
Human gut - healthy	Human gut microbiome viewed across age and geography (WGS)	4461160.3	Gordon, J.	http://metagenomics.anl.gov/metagenomics.cgi?page=MetagenomeProject&project=98	2012	http://metagenomics.anl.gov/linkin.cgi?metagenome=4461160.3
Human gut - healthy	Human gut microbiome viewed across age and geography (WGS)	4461161.3	Gordon, J.	http://metagenomics.anl.gov/metagenomics.cgi?page=MetagenomeProject&project=98	2012	http://metagenomics.anl.gov/linkin.cgi?metagenome=4461161.3
Human gut - healthy	Human gut microbiome viewed across age and geography (WGS)	4461162.3	Gordon, J.	http://metagenomics.anl.gov/metagenomics.cgi?page=MetagenomeProject&project=98	2012	http://metagenomics.anl.gov/linkin.cgi?metagenome=4461162.3
Human gut - healthy	Human gut microbiome viewed across age and geography (WGS)	4461163.3	Gordon, J.	http://metagenomics.anl.gov/metagenomics.cgi?page=MetagenomeProject&project=98	2012	http://metagenomics.anl.gov/linkin.cgi?metagenome=4461163.3
Human gut - healthy	Human gut microbiome viewed across age and geography (WGS)	4461164.3	Gordon, J.	http://metagenomics.anl.gov/metagenomics.cgi?page=MetagenomeProject&project=98	2012	http://metagenomics.anl.gov/linkin.cgi?metagenome=4461164.3

Human gut - healthy	Human gut microbiome viewed across age and geography (WGS)	4461165.3	Gordon, J.	http://metagenomics.anl.gov/metagenomics.cgi?page=MetagenomeProject&project=98	2012	http://metagenomics.anl.gov/linkin.cgi?metagenome=4461165.3
Human gut - healthy	Human gut microbiome viewed across age and geography (WGS)	4461166.3	Gordon, J.	http://metagenomics.anl.gov/metagenomics.cgi?page=MetagenomeProject&project=98	2012	http://metagenomics.anl.gov/linkin.cgi?metagenome=4461166.3
Human gut - healthy	Human gut microbiome viewed across age and geography (WGS)	4461167.3	Gordon, J.	http://metagenomics.anl.gov/metagenomics.cgi?page=MetagenomeProject&project=98	2012	http://metagenomics.anl.gov/linkin.cgi?metagenome=4461167.3
Human gut - healthy	Human gut microbiome viewed across age and geography (WGS)	4461168.3	Gordon, J.	http://metagenomics.anl.gov/metagenomics.cgi?page=MetagenomeProject&project=98	2012	http://metagenomics.anl.gov/linkin.cgi?metagenome=4461168.3
Human gut - healthy	Human gut microbiome viewed across age and geography (WGS)	4461169.3	Gordon, J.	http://metagenomics.anl.gov/metagenomics.cgi?page=MetagenomeProject&project=98	2012	http://metagenomics.anl.gov/linkin.cgi?metagenome=4461169.3
Human gut - healthy	Human gut microbiome viewed across age and geography (WGS)	4461170.3	Gordon, J.	http://metagenomics.anl.gov/metagenomics.cgi?page=MetagenomeProject&project=98	2012	http://metagenomics.anl.gov/linkin.cgi?metagenome=4461170.3
Human gut - healthy	Human gut microbiome viewed across age and geography (WGS)	4461171.3	Gordon, J.	http://metagenomics.anl.gov/metagenomics.cgi?page=MetagenomeProject&project=98	2012	http://metagenomics.anl.gov/linkin.cgi?metagenome=4461171.3
Human gut - healthy	Human gut microbiome viewed across age and geography (WGS)	4461172.3	Gordon, J.	http://metagenomics.anl.gov/metagenomics.cgi?page=MetagenomeProject&project=98	2012	http://metagenomics.anl.gov/linkin.cgi?metagenome=4461172.3
Human gut - healthy	Human gut microbiome viewed across age and geography (WGS)	4461173.3	Gordon, J.	http://metagenomics.anl.gov/metagenomics.cgi?page=MetagenomeProject&project=98	2012	http://metagenomics.anl.gov/linkin.cgi?metagenome=4461173.3
Human gut - healthy	Human gut microbiome viewed across age and geography (WGS)	4461174.3	Gordon, J.	http://metagenomics.anl.gov/metagenomics.cgi?page=MetagenomeProject&project=98	2012	http://metagenomics.anl.gov/linkin.cgi?metagenome=4461174.3
Human gut - healthy	Human gut microbiome viewed across age and geography (WGS)	4461175.3	Gordon, J.	http://metagenomics.anl.gov/metagenomics.cgi?page=MetagenomeProject&project=98	2012	http://metagenomics.anl.gov/linkin.cgi?metagenome=4461175.3
Human gut - healthy	Human gut microbiome viewed across age and geography (WGS)	4461176.3	Gordon, J.	http://metagenomics.anl.gov/metagenomics.cgi?page=MetagenomeProject&project=98	2012	http://metagenomics.anl.gov/linkin.cgi?metagenome=4461176.3
Human gut - healthy	Human gut microbiome viewed across age and geography (WGS)	4461177.3	Gordon, J.	http://metagenomics.anl.gov/metagenomics.cgi?page=MetagenomeProject&project=98	2012	http://metagenomics.anl.gov/linkin.cgi?metagenome=4461177.3
Human gut - healthy	Human gut microbiome viewed across age and geography (WGS)	4461178.3	Gordon, J.	http://metagenomics.anl.gov/metagenomics.cgi?page=MetagenomeProject&project=98	2012	http://metagenomics.anl.gov/linkin.cgi?metagenome=4461178.3
Human gut - healthy	Human gut microbiome viewed across age and geography (WGS)	4461179.3	Gordon, J.	http://metagenomics.anl.gov/metagenomics.cgi?page=MetagenomeProject&project=98	2012	http://metagenomics.anl.gov/linkin.cgi?metagenome=4461179.3
Human gut - healthy	Human gut microbiome viewed across age and geography (WGS)	4461180.3	Gordon, J.	http://metagenomics.anl.gov/metagenomics.cgi?page=MetagenomeProject&project=98	2012	http://metagenomics.anl.gov/linkin.cgi?metagenome=4461180.3
Human gut - healthy	Human gut microbiome viewed across age and geography (WGS)	4461181.3	Gordon, J.	http://metagenomics.anl.gov/metagenomics.cgi?page=MetagenomeProject&project=98	2012	http://metagenomics.anl.gov/linkin.cgi?metagenome=4461181.3
Human gut - healthy	Human gut microbiome viewed across age and geography (WGS)	4461182.3	Gordon, J.	http://metagenomics.anl.gov/metagenomics.cgi?page=MetagenomeProject&project=98	2012	http://metagenomics.anl.gov/linkin.cgi?metagenome=4461182.3
Human gut - healthy	Human gut microbiome viewed across age and geography (WGS)	4461183.3	Gordon, J.	http://metagenomics.anl.gov/metagenomics.cgi?page=MetagenomeProject&project=98	2012	http://metagenomics.anl.gov/linkin.cgi?metagenome=4461183.3
Human gut - healthy	Human gut microbiome viewed across age and geography (WGS)	4461184.3	Gordon, J.	http://metagenomics.anl.gov/metagenomics.cgi?page=MetagenomeProject&project=98	2012	http://metagenomics.anl.gov/linkin.cgi?metagenome=4461184.3
Human gut - healthy	Human gut microbiome viewed across age and geography (WGS)	4461185.3	Gordon, J.	http://metagenomics.anl.gov/metagenomics.cgi?page=MetagenomeProject&project=98	2012	http://metagenomics.anl.gov/linkin.cgi?metagenome=4461185.3
Human gut - healthy	Human gut microbiome viewed across age and geography (WGS)	4461186.3	Gordon, J.	http://metagenomics.anl.gov/metagenomics.cgi?page=MetagenomeProject&project=98	2012	http://metagenomics.anl.gov/linkin.cgi?metagenome=4461186.3
Human gut - healthy	Human gut microbiome viewed across age and geography (WGS)	4461187.3	Gordon, J.	http://metagenomics.anl.gov/metagenomics.cgi?page=MetagenomeProject&project=98	2012	http://metagenomics.anl.gov/linkin.cgi?metagenome=4461187.3
Human gut - healthy	Human gut microbiome viewed across age and geography (WGS)	4461188.3	Gordon, J.	http://metagenomics.anl.gov/metagenomics.cgi?page=MetagenomeProject&project=98	2012	http://metagenomics.anl.gov/linkin.cgi?metagenome=4461188.3
Human gut - healthy	Human gut microbiome viewed across age and geography (WGS)	4461189.3	Gordon, J.	http://metagenomics.anl.gov/metagenomics.cgi?page=MetagenomeProject&project=98	2012	http://metagenomics.anl.gov/linkin.cgi?metagenome=4461189.3
Human gut - healthy	Human gut microbiome viewed across age and geography (WGS)	4461190.3	Gordon, J.	http://metagenomics.anl.gov/metagenomics.cgi?page=MetagenomeProject&project=98	2012	http://metagenomics.anl.gov/linkin.cgi?metagenome=4461190.3
Human gut - healthy	Human gut microbiome viewed across age and geography (WGS)	4461191.3	Gordon, J.	http://metagenomics.anl.gov/metagenomics.cgi?page=MetagenomeProject&project=98	2012	http://metagenomics.anl.gov/linkin.cgi?metagenome=4461191.3
Human gut - healthy	Human gut microbiome viewed across age and geography (WGS)	4461192.3	Gordon, J.	http://metagenomics.anl.gov/metagenomics.cgi?page=MetagenomeProject&project=98	2012	http://metagenomics.anl.gov/linkin.cgi?metagenome=4461192.3
Human gut - healthy	Human gut microbiome viewed across age and geography (WGS)	4461193.3	Gordon, J.	http://metagenomics.anl.gov/metagenomics.cgi?page=MetagenomeProject&project=98	2012	http://metagenomics.anl.gov/linkin.cgi?metagenome=4461193.3
Human gut - healthy	Human gut microbiome viewed across age and geography (WGS)	4461195.3	Gordon, J.	http://metagenomics.anl.gov/metagenomics.cgi?page=MetagenomeProject&project=98	2012	http://metagenomics.anl.gov/linkin.cgi?metagenome=4461195.3
Human gut - healthy	Human gut microbiome viewed across age and geography (WGS)	4461196.3	Gordon, J.	http://metagenomics.anl.gov/metagenomics.cgi?page=MetagenomeProject&project=98	2012	http://metagenomics.anl.gov/linkin.cgi?metagenome=4461196.3
Human gut - healthy	Human gut microbiome viewed across age and geography (WGS)	4461197.3	Gordon, J.	http://metagenomics.anl.gov/metagenomics.cgi?page=MetagenomeProject&project=98	2012	http://metagenomics.anl.gov/linkin.cgi?metagenome=4461197.3
Human gut - healthy	Human gut microbiome viewed across age and geography (WGS)	4461198.3	Gordon, J.	http://metagenomics.anl.gov/metagenomics.cgi?page=MetagenomeProject&project=98	2012	http://metagenomics.anl.gov/linkin.cgi?metagenome=4461198.3
Human gut - healthy	Human gut microbiome viewed across age and geography (WGS)	4461199.3	Gordon, J.	http://metagenomics.anl.gov/metagenomics.cgi?page=MetagenomeProject&project=98	2012	http://metagenomics.anl.gov/linkin.cgi?metagenome=4461199.3
Human gut - healthy	Human gut microbiome viewed across age and geography (WGS)	4461200.3	Gordon, J.	http://metagenomics.anl.gov/metagenomics.cgi?page=MetagenomeProject&project=98	2012	http://metagenomics.anl.gov/linkin.cgi?metagenome=4461200.3
Human gut - healthy	Human gut microbiome viewed across age and geography (WGS)	4461201.3	Gordon, J.	http://metagenomics.anl.gov/metagenomics.cgi?page=MetagenomeProject&project=98	2012	http://metagenomics.anl.gov/linkin.cgi?metagenome=4461201.3
Human gut - healthy	Human gut microbiome viewed across age and geography (WGS)	4461202.3	Gordon, J.	http://metagenomics.anl.gov/metagenomics.cgi?page=MetagenomeProject&project=98	2012	http://metagenomics.anl.gov/linkin.cgi?metagenome=4461202.3
Human gut - healthy	Human gut microbiome viewed across age and geography (WGS)	4461203.3	Gordon, J.	http://metagenomics.anl.gov/metagenomics.cgi?page=MetagenomeProject&project=98	2012	http://metagenomics.anl.gov/linkin.cgi?metagenome=4461203.3
Human gut - healthy	Human gut microbiome viewed across age and geography (WGS)	4461204.3	Gordon, J.	http://metagenomics.anl.gov/metagenomics.cgi?page=MetagenomeProject&project=98	2012	http://metagenomics.anl.gov/linkin.cgi?metagenome=4461204.3
Human gut - healthy	Human gut microbiome viewed across age and geography (WGS)	4461205.3	Gordon, J.	http://metagenomics.anl.gov/metagenomics.cgi?page=MetagenomeProject&project=98	2012	http://metagenomics.anl.gov/linkin.cgi?metagenome=4461205.3
Human gut - healthy	Human gut microbiome viewed across age and geography (WGS)	4461206.3	Gordon, J.	http://metagenomics.anl.gov/metagenomics.cgi?page=MetagenomeProject&project=98	2012	http://metagenomics.anl.gov/linkin.cgi?metagenome=4461206.3
Human gut - healthy	Human gut microbiome viewed across age and geography (WGS)	4461207.3	Gordon, J.	http://metagenomics.anl.gov/metagenomics.cgi?page=MetagenomeProject&project=98	2012	http://metagenomics.anl.gov/linkin.cgi?metagenome=4461207.3
Human gut - healthy	Human gut microbiome viewed across age and geography (WGS)	4461208.3	Gordon, J.	http://metagenomics.anl.gov/metagenomics.cgi?page=MetagenomeProject&project=98	2012	http://metagenomics.anl.gov/linkin.cgi?metagenome=4461208.3
Human gut - healthy	Human gut microbiome viewed across age and geography (WGS)	4461210.3	Gordon, J.	http://metagenomics.anl.gov/metagenomics.cgi?page=MetagenomeProject&project=98	2012	http://metagenomics.anl.gov/linkin.cgi?metagenome=4461210.3
Human gut - healthy	Human gut microbiome viewed across age and geography (WGS)	4461211.3	Gordon, J.	http://metagenomics.anl.gov/metagenomics.cgi?page=MetagenomeProject&project=98	2012	http://metagenomics.anl.gov/linkin.cgi?metagenome=4461211.3
Human gut - healthy	Human gut microbiome viewed across age and geography (WGS)	4461212.3	Gordon, J.	http://metagenomics.anl.gov/metagenomics.cgi?page=MetagenomeProject&project=98	2012	http://metagenomics.anl.gov/linkin.cgi?metagenome=4461212.3
Human gut - healthy	Human gut microbiome viewed across age and geography (WGS)	4461213.3	Gordon, J.	http://metagenomics.anl.gov/metagenomics.cgi?page=MetagenomeProject&project=98	2012	http://metagenomics.anl.gov/linkin.cgi?metagenome=4461213.3
Human gut - healthy	Human gut microbiome viewed across age and geography (WGS)	4461214.3	Gordon, J.	http://metagenomics.anl.gov/metagenomics.cgi?page=MetagenomeProject&project=98	2012	http://metagenomics.anl.gov/linkin.cgi?metagenome=4461214.3
Human gut - healthy	Human gut microbiome viewed across age and geography (WGS)	4461215.3	Gordon, J.	http://metagenomics.anl.gov/metagenomics.cgi?page=MetagenomeProject&project=98	2012	http://metagenomics.anl.gov/linkin.cgi?metagenome=4461215.3
Human gut - healthy	Human gut microbiome viewed across age and geography (WGS)	4461216.3	Gordon, J.	http://metagenomics.anl.gov/metagenomics.cgi?page=MetagenomeProject&project=98	2012	http://metagenomics.anl.gov/linkin.cgi?metagenome=4461216.3
Human gut - healthy	Human gut microbiome viewed across age and geography (WGS)	4461217.3	Gordon, J.	http://metagenomics.anl.gov/metagenomics.cgi?page=MetagenomeProject&project=98	2012	http://metagenomics.anl.gov/linkin.cgi?metagenome=4461217.3
Human gut - healthy	Human gut microbiome viewed across age and geography (WGS)	4461218.3	Gordon, J.	http://metagenomics.anl.gov/metagenomics.cgi?page=MetagenomeProject&project=98	2012	http://metagenomics.anl.gov/linkin.cgi?metagenome=4461218.3
Human gut - healthy	Human gut microbiome viewed across age and geography (WGS)	4461219.3	Gordon, J.	http://metagenomics.anl.gov/metagenomics.cgi?page=MetagenomeProject&project=98	2012	http://metagenomics.anl.gov/linkin.cgi?metagenome=4461219.3
Human gut - healthy	Human gut microbiome viewed across age and geography (WGS)	4461220.3	Gordon, J.	http://metagenomics.anl.gov/metagenomics.cgi?page=MetagenomeProject&project=98	2012	http://metagenomics.anl.gov/linkin.cgi?metagenome=4461220.3
Human gut - healthy	Human gut microbiome viewed across age and geography (WGS)	4461221.3	Gordon, J.	http://metagenomics.anl.gov/metagenomics.cgi?page=MetagenomeProject&project=98	2012	http://metagenomics.anl.gov/linkin.cgi?metagenome=4461221.3
Human gut - healthy	Human gut microbiome viewed across age and geography (WGS)	4461222.3	Gordon, J.	http://metagenomics.anl.gov/metagenomics.cgi?page=MetagenomeProject&project=98	2012	http://metagenomics.anl.gov/linkin.cgi?metagenome=4461222.3
Human gut - healthy	Human gut microbiome viewed across age and geography (WGS)	4461223.3	Gordon, J.	http://metagenomics.anl.gov/metagenomics.cgi?page=MetagenomeProject&project=98	2012	http://metagenomics.anl.gov/linkin.cgi?metagenome=4461223.3
Human gut - healthy	Human gut microbiome viewed across age and geography (WGS)	4461224.3	Gordon, J.	http://metagenomics.anl.gov/metagenomics.cgi?page=MetagenomeProject&project=98	2012	http://metagenomics.anl.gov/linkin.cgi?metagenome=4461224.3
Human gut - healthy	Human gut microbiome viewed across age and geography (WGS)	4461225.3	Gordon, J.	http://metagenomics.anl.gov/metagenomics.cgi?page=MetagenomeProject&project=98	2012	http://metagenomics.anl.gov/linkin.cgi?metagenome=4461225.3
Human gut - healthy	Human gut microbiome viewed across age and geography (WGS)	4461226.3	Gordon, J.	http://metagenomics.anl.gov/metagenomics.cgi?page=MetagenomeProject&project=98	2012	http://metagenomics.anl.gov/linkin.cgi?metagenome=4461226.3
Human gut - healthy	Human gut microbiome viewed across age and geography (WGS)	4461227.3	Gordon, J.	http://metagenomics.anl.gov/metagenomics.cgi?page=MetagenomeProject&project=98	2012	http://metagenomics.anl.gov/linkin.cgi?metagenome=4461227.3
Human gut - healthy	Human gut microbiome viewed across age and geography (WGS)	4461228.3	Gordon, J.	http://metagenomics.anl.gov/metagenomics.cgi?page=MetagenomeProject&project=98	2012	http://metagenomics.anl.gov/linkin.cgi?metagenome=4461228.3
Human gut - healthy	Human gut microbiome viewed across age and geography (WGS)	4461229.3	Gordon, J.	http://metagenomics.anl.gov/metagenomics.cgi?page=MetagenomeProject&project=98	2012	http://metagenomics.anl.gov/linkin.cgi?metagenome=4461229.3
Human gut - healthy	Healthy human gut microbial metagenomic study	4525093.3	Hattori, M.	http://metagenomics.anl.gov/metagenomics.cgi?page=MetagenomeProject&project=4778	2007	http://metagenomics.anl.gov/linkin.cgi?metagenome=4525093.3
Human gut - healthy	Healthy human gut microbial metagenomic study	4525311.3	Hattori, M.	http://metagenomics.anl.gov/metagenomics.cgi?page=MetagenomeProject&project=4778	2007	http://metagenomics.anl.gov/linkin.cgi?metagenome=4525311.3
Human gut - healthy	Healthy human gut microbial metagenomic study	4525312.3	Hattori, M.	http://metagenomics.anl.gov/metagenomics.cgi?page=MetagenomeProject&project=4778	2007	http://metagenomics.anl.gov/linkin.cgi?metagenome=4525312.3
Human gut - healthy	Healthy human gut microbial metagenomic study	4525314.3	Hattori, M.	http://metagenomics.anl.gov/metagenomics.cgi?page=MetagenomeProject&project=4778	2007	http://metagenomics.anl.gov/linkin.cgi?metagenome=4525314.3
Human gut - healthy	Healthy human gut microbial metagenomic study	4525315.3	Hattori, M.	http://metagenomics.anl.gov/metagenomics.cgi?page=MetagenomeProject&project=4778	2007	http://metagenomics.anl.gov/linkin.cgi?metagenome=4525315.3
Human gut - healthy	Healthy human gut microbial metagenomic study	4525323.3	Hattori, M.	http://metagenomics.anl.gov/metagenomics.cgi?page=MetagenomeProject&project=4778	2007	http://metagenomics.anl.gov/linkin.cgi?metagenome=4525323.3
Human gut - healthy	Healthy human gut microbial metagenomic study	4525324.3	Hattori, M.	http://metagenomics.anl.gov/metagenomics.cgi?page=MetagenomeProject&project=4778	2007	http://metagenomics.anl.gov/linkin.cgi?metagenome=4525324.3
Human gut - healthy	Human-gut (Caroline De Tender)	4562213.3	De Tender, C.	http://metagenomics.anl.gov/metagenomics.cgi?page=MetagenomeProject&project=8939	2009	http://metagenomics.anl.gov/linkin.cgi?metagenome=4562213.3
Human gut - healthy	Human-gut (Caroline De Tender)	4562214.3	De Tender, C.	http://metagenomics.anl.gov/metagenomics.cgi?page=MetagenomeProject&project=8939	2009	http://metagenomics.anl.gov/linkin.cgi?metagenome=4562214.3
Human gut - healthy	Human-gut (Caroline De Tender)	4562215.3	De Tender, C.	http://metagenomics.anl.gov/metagenomics.cgi?page=MetagenomeProject&project=8939	2009	http://metagenomics.anl.gov/linkin.cgi?metagenome=4562215.3
Human gut - healthy	Human-gut (Caroline De Tender)	4562216.3	De Tender, C.	http://metagenomics.anl.gov/metagenomics.cgi?page=MetagenomeProject&project=8939	2009	http://metagenomics.anl.gov/linkin.cgi?metagenome=4562216.3
Human gut - stress	Host Remodeling of the Gut Microbiome and Metabolic Changes during Pregnancy	4466148.3	Ley, R.	http://metagenomics.anl.gov/metagenomics.cgi?page=MetagenomeProject&project=265	2008	http://metagenomics.anl.gov/linkin.cgi?metagenome=4466148.3
Human gut - stress	Host Remodeling of the Gut Microbiome and Metabolic Changes during Pregnancy	4466149.3	Ley, R.	http://metagenomics.anl.gov/metagenomics.cgi?page=MetagenomeProject&project=265	2008	http://metagenomics.anl.gov/linkin.cgi?metagenome=4466149.3
Human gut - stress	Host Remodeling of the Gut Microbiome and Metabolic Changes during Pregnancy	4466163.3	Ley, R.	http://metagenomics.anl.gov/metagenomics.cgi?page=MetagenomeProject&project=265	2008	http://metagenomics.anl.gov/linkin.cgi?metagenome=4466163.3
Human gut - stress	Host Remodeling of the Gut Microbiome and Metabolic Changes during Pregnancy	4466188.3	Ley, R.	http://metagenomics.anl.gov/metagenomics.cgi?page=MetagenomeProject&project=265	2008	http://metagenomics.anl.gov/linkin.cgi?metagenome=4466188.3
Human gut - stress	Host Remodeling of the Gut Microbiome and Metabolic Changes during Pregnancy	4466194.3	Ley, R.	http://metagenomics.anl.gov/metagenomics.cgi?page=MetagenomeProject&project=265	2008	http://metagenomics.anl.gov/linkin.cgi?metagenome=4466194.3
Human gut - stress	Host Remodeling of the Gut Microbiome and Metabolic Changes during Pregnancy	4466257.3	Ley, R.	http://metagenomics.anl.gov/metagenomics.cgi?page=MetagenomeProject&project=265	2008	http://metagenomics.anl.gov/linkin.cgi?metagenome=4466257.3
Human gut - stress	Host Remodeling of the Gut Microbiome and Metabolic Changes during Pregnancy	4466296.3	Ley, R.	http://metagenomics.anl.gov/metagenomics.cgi?page=MetagenomeProject&project=265	2008	http://metagenomics.anl.gov/linkin.cgi?metagenome=4466296.3
Human gut - stress	Host Remodeling of the Gut Microbiome and Metabolic Changes during Pregnancy	4466602.3	Ley, R.	http://metagenomics.anl.gov/metagenomics.cgi?page=MetagenomeProject&project=265	2008	http://metagenomics.anl.gov/linkin.cgi?metagenome=4466602.3
Human gut - stress	Host Remodeling of the Gut Microbiome and Metabolic Changes during Pregnancy	4467109.3	Ley, R.	http://metagenomics.anl.gov/metagenomics.cgi?page=MetagenomeProject&project=265	2008	http://metagenomics.anl.gov/linkin.cgi?metagenome=4467109.3
Human gut - stress	Host Remodeling of the Gut Microbiome and Metabolic Changes during Pregnancy	4469858.3	Ley, R.	http://metagenomics.anl.gov/metagenomics.cgi?page=MetagenomeProject&project=265	2008	http://metagenomics.anl.gov/linkin.cgi?metagenome=4469858.3
Human gut - stress	Host Remodeling of the Gut Microbiome and Metabolic Changes during Pregnancy	4474351.3	Ley, R.	http://metagenomics.anl.gov/metagenomics.cgi?page=MetagenomeProject&project=265	2008	http://metagenomics.anl.gov/linkin.cgi?metagenome=4474351.3
Human gut - stress	Host Remodeling of the Gut Microbiome and Metabolic Changes during Pregnancy	4474352.3	Ley, R.	http://metagenomics.anl.gov/metagenomics.cgi?page=MetagenomeProject&project=265	2008	http://metagenomics.anl.gov/linkin.cgi?metagenome=4474352.3
Human gut - stress	Host Remodeling of the Gut Microbiome and Metabolic Changes during Pregnancy	4474355.3	Ley, R.	http://metagenomics.anl.gov/metagenomics.cgi?page=MetagenomeProject&project=265	2008	http://metagenomics.anl.gov/linkin.cgi?metagenome=4474355.3
Human gut - stress	Host Remodeling of the Gut Microbiome and Metabolic Changes during Pregnancy	4474357.3	Ley, R.	http://metagenomics.anl.gov/metagenomics.cgi?page=MetagenomeProject&project=265	2008	http://metagenomics.anl.gov/linkin.cgi?metagenome=4474357.3
Human gut - stress	Host Remodeling of the Gut Microbiome and Metabolic Changes during Pregnancy	4474358.3	Ley, R.	http://metagenomics.anl.gov/metagenomics.cgi?page=MetagenomeProject&project=265	2008	http://metagenomics.anl.gov/linkin.cgi?metagenome=4474358.3
Human gut - stress	Host Remodeling of the Gut Microbiome and Metabolic Changes during Pregnancy	4474359.3	Ley, R.	http://metagenomics.anl.gov/metagenomics.cgi?page=MetagenomeProject&project=265	2008	http://metagenomics.anl.gov/linkin.cgi?metagenome=4474359.3
Human gut - stress	Host Remodeling of the Gut Microbiome and Metabolic Changes during Pregnancy	4474360.3	Ley, R.	http://metagenomics.anl.gov/metagenomics.cgi?page=MetagenomeProject&project=265	2008	http://metagenomics.anl.gov/linkin.cgi?metagenome=4474360.3
Human gut - stress	Host Remodeling of the Gut Microbiome and Metabolic Changes during Pregnancy	4474361.3	Ley, R.	http://metagenomics.anl.gov/metagenomics.cgi?page=MetagenomeProject&project=265	2008	http://metagenomics.anl.gov/linkin.cgi?metagenome=4474361.3
Human gut - stress	Host Remodeling of the Gut Microbiome and Metabolic Changes during Pregnancy	4474369.3	Ley, R.	http://metagenomics.anl.gov/metagenomics.cgi?page=MetagenomeProject&project=265	2008	http://metagenomics.anl.gov/linkin.cgi?metagenome=4474369.3
Human gut - stress	Host Remodeling of the Gut Microbiome and Metabolic Changes during Pregnancy	4474805.3	Ley, R.	http://metagenomics.anl.gov/metagenomics.cgi?page=MetagenomeProject&project=265	2008	http://metagenomics.anl.gov/linkin.cgi?metagenome=4474805.3
Human gut - stress	UMC Utrecht, NL, ICU patient gut microbiome	4508944.3	Buelow, E.	http://metagenomics.anl.gov/metagenomics.cgi?page=MetagenomeProject&project=2851	2012	http://metagenomics.anl.gov/linkin.cgi?metagenome=4508944.3
Human gut - stress	UMC Utrecht, NL, ICU patient gut microbiome	4508945.3	Buelow, E.	http://metagenomics.anl.gov/metagenomics.cgi?page=MetagenomeProject&project=2851	2012	http://metagenomics.anl.gov/linkin.cgi?metagenome=4508945.3
Human gut - stress	UMC Utrecht, NL, ICU patient gut microbiome	4508946.3	Buelow, E.	http://metagenomics.anl.gov/metagenomics.cgi?page=MetagenomeProject&project=2851	2012	http://metagenomics.anl.gov/linkin.cgi?metagenome=4508946.3
Human gut - stress	UMC Utrecht, NL, ICU patient gut microbiome	4508947.3	Buelow, E.	http://metagenomics.anl.gov/metagenomics.cgi?page=MetagenomeProject&project=2851	2012	http://metagenomics.anl.gov/linkin.cgi?metagenome=4508947.3
Human gut - stress	Functional metagenomic profiling of intestinal microbiome in extreme ageing	4507016.3	Rampelli, S.	http://metagenomics.anl.gov/metagenomics.cgi?page=MetagenomeProject&project=2558	2013	http://metagenomics.anl.gov/linkin.cgi?metagenome=4507016.3
Human gut - stress	Functional metagenomic profiling of intestinal microbiome in extreme ageing	4508973.3	Rampelli, S.	http://metagenomics.anl.gov/metagenomics.cgi?page=MetagenomeProject&project=2558	2013	http://metagenomics.anl.gov/linkin.cgi?metagenome=4508973.3
Human gut - stress	Functional metagenomic profiling of intestinal microbiome in extreme ageing	4508975.3	Rampelli, S.	http://metagenomics.anl.gov/metagenomics.cgi?page=MetagenomeProject&project=2558	2013	http://metagenomics.anl.gov/linkin.cgi?metagenome=4508975.3
Human gut - stress	Functional metagenomic profiling of intestinal microbiome in extreme ageing	4508976.3	Rampelli, S.	http://metagenomics.anl.gov/metagenomics.cgi?page=MetagenomeProject&project=2558	2013	http://metagenomics.anl.gov/linkin.cgi?metagenome=4508976.3
Human gut - stress	Functional metagenomic profiling of intestinal microbiome in extreme ageing	4508977.3	Rampelli, S.	http://metagenomics.anl.gov/metagenomics.cgi?page=MetagenomeProject&project=2558	2013	http://metagenomics.anl.gov/linkin.cgi?metagenome=4508977.3
Human gut - stress	Functional metagenomic profiling of intestinal microbiome in extreme ageing	4508978.3	Rampelli, S.	http://metagenomics.anl.gov/metagenomics.cgi?page=MetagenomeProject&project=2558	2013	http://metagenomics.anl.gov/linkin.cgi?metagenome=4508978.3
Human gut - stress	Functional metagenomic profiling of intestinal microbiome in extreme ageing	4508979.3	Rampelli, S.	http://metagenomics.anl.gov/metagenomics.cgi?page=MetagenomeProject&project=2558	2013	http://metagenomics.anl.gov/linkin.cgi?metagenome=4508979.3
Human gut - stress	Functional metagenomic profiling of intestinal microbiome in extreme ageing	4508980.3	Rampelli, S.	http://metagenomics.anl.gov/metagenomics.cgi?page=MetagenomeProject&project=2558	2013	http://metagenomics.anl.gov/linkin.cgi?metagenome=4508980.3
